# Phagocytosis of environmental or metabolic crystalline particles induces cytotoxicity by triggering necroptosis across a broad range of particle size and shape

**DOI:** 10.1038/s41598-017-15804-9

**Published:** 2017-11-14

**Authors:** Mohsen Honarpisheh, Orestes Foresto-Neto, Jyaysi Desai, Stefanie Steiger, Lidia Anguiano Gómez, Bastian Popper, Peter Boor, Hans-Joachim Anders, Shrikant R. Mulay

**Affiliations:** 1 Medizinische Klinik und Poliklinik IV, Klinikum der Universität, München, Munich, 80336 Germany; 2 Biomedical Center (BMC), Department for Cell Biology, Ludwig-Maximilians University, Munich, 82152 Germany; 3 Institute of Pathology & Dept. of Nephrology, University Clinic of RWTH Aachen, Aachen, 52074 Germany

## Abstract

In crystallopathies, crystals or crystalline particles of environmental and metabolic origin deposit within tissues, induce inflammation, injury and cell death and eventually lead to organ-failure. The NLRP3-inflammasome is involved in mediating crystalline particles-induced inflammation, but pathways leading to cell death are still unknown. Here, we have used broad range of intrinsic and extrinsic crystal- or crystalline particle-sizes and shapes, e.g. calcium phosphate, silica, titanium dioxide, cholesterol, calcium oxalate, and monosodium urate. As kidney is commonly affected by crystallopathies, we used human and murine renal tubular cells as a model system. We showed that all of the analysed crystalline particles induce caspase-independent cell death. Deficiency of MLKL, siRNA knockdown of RIPK3, or inhibitors of necroptosis signaling e.g. RIPK-1 inhibitor necrostatin-1s, RIPK3 inhibitor dabrafenib, and MLKL inhibitor necrosulfonamide, partially protected tubular cells from crystalline particles cytotoxicity. Furthermore, we identify phagocytosis of crystalline particles as an upstream event in their cytotoxicity since a phagocytosis inhibitor, cytochalasin D, prevented their cytotoxicity. Taken together, our data confirmed the involvement of necroptosis as one of the pathways leading to cell death in crystallopathies. Our data identified RIPK-1, RIPK3, and MLKL as molecular targets to limit tissue injury and organ failure in crystallopathies.

## Introduction

Crystals of intrinsic or extrinsic origin induce inflammation and tissue injury when deposited inside the body triggering diverse medical disorders termed as “crystallopathies”^1^ e.g. occupational dust-induced lung injuries^1–3^ (silica crystals and titanium dioxide (TiO_2_) nanoparticles), various forms of crystal nephropathies^1,4,5^ (crystals of calcium oxalate (CaOx), monosodium urate (MSU), and calcium phosphate (CaP)), gouty arthritis^1,6^ (MSU crystals), atherosclerosis^1,7^ (cholesterol crystals). These crystallopathies are characterized by crystal-induced acute necroinflammation^1,8,9^. Although the capability of crystals and crystalline materials to induce NOD-like receptor protein (NLRP)-3 inflammasome-mediated interleukin (IL)-1β, IL-18 release, and subsequent inflammation gained importance as a major pathomechanism of these crystallopathies^10^, their cytotoxic effects have remained poorly explored. Crystals induce cell necrosis rather than apoptosis^11,12^. However, it has remained unclear whether crystal cytotoxicity is a consequence of passive or regulated necrosis until recently when we reported that intrinsic CaOx crystal deposition induces receptor interacting protein kinase-3 (RIPK3) – mixed lineage kinase domain-like (MLKL)-mediated necroptosis in tubular epithelial cells during acute oxalate nephropathy^8^. Since, CaOx crystals can also activate the NLRP3 inflammasome^13^ in a similar manner as it is reported for crystals of silica^14,15^, cholesterol^16^, MSU^17^, CaP^18^ and TiO_2_ nanoparticles^19^, therefore, we here hypothesized that both environmental (silica, CaP, TiO_2_) and metabolic (cholesterol, MSU, CaP, CaOx) crystals induce RIPK3-MLKL-mediated necroptosis in human cells.

## Results

### Different sizes and shapes of environmental or metabolic crystalline particles induce cell death

**Figure 1:**
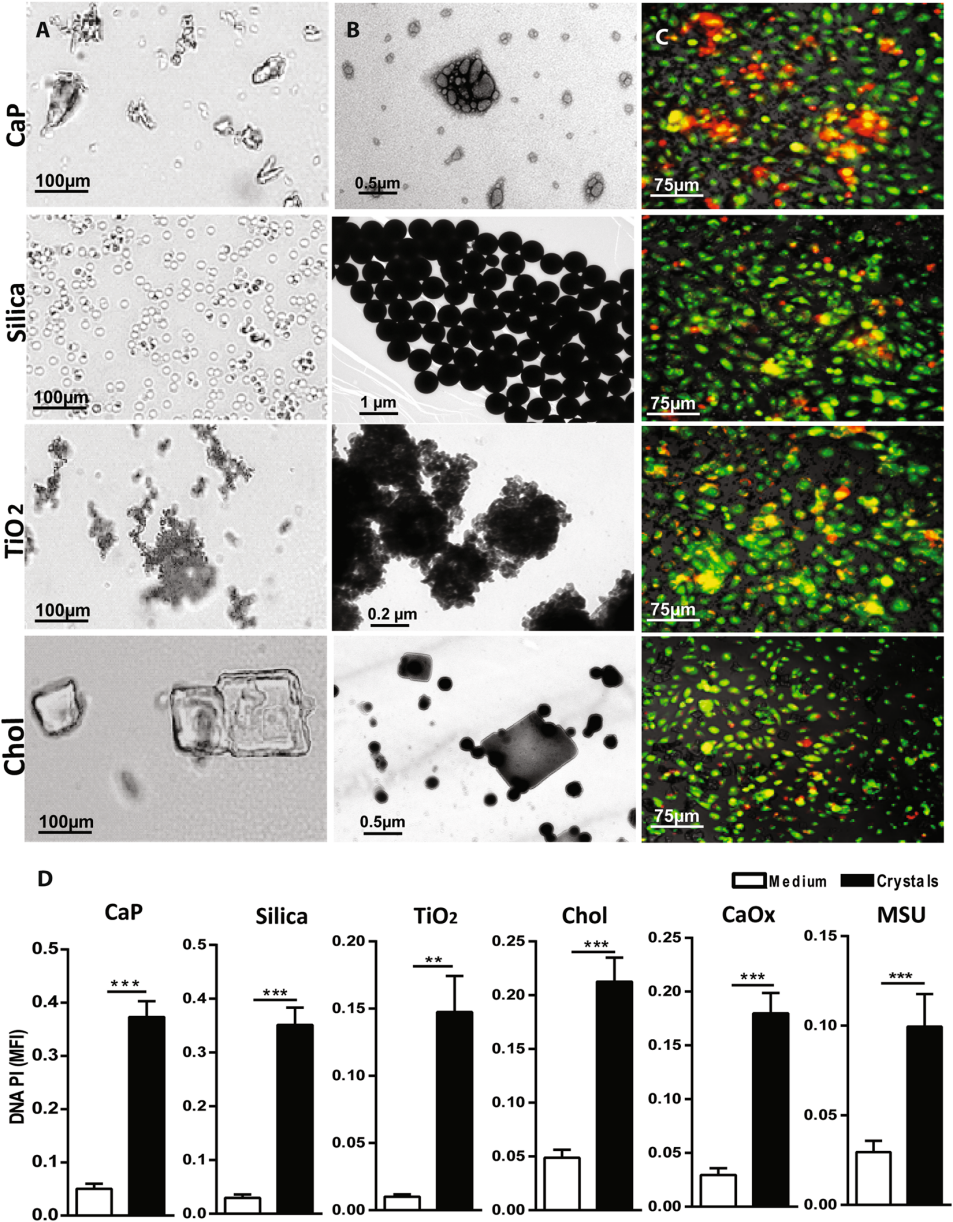
Different sizes and shapes of crystals or crystalline particles induce cell death in HK-2 cells. (**A**,**B**) Crystals of CaP, silica, cholesterol, and TiO_2_ nanoparticles were visualized by light microscopy (**A**) and TEM (**B**) Note the different sizes and shapes of all crystals. (**C**) HK-2 cells were exposed to CaP (1 mg/ml), silica (1 mg/ml), TiO_2_ (0.5 mg/ml), cholesterol (3 mg/ml), CaOx (1 mg/ml), and MSU (0.5 mg/ml) for 24 hrs. Cell death was visualized by PI stain (red color). Acridine orange (green color) stained live cells. PI images were converted into black and white image for better visualization using ImageJ software. (**D**) Quantification of DNA-PI mean fluorescence intensity (MFI). Data are expressed as mean ± SEM from three independent experiments.

Whether environment crystals can induce cell death, and whether their sizes and shapes have an impact on their cytotoxicity, is not clear. To address these questions, we studied a broad range of environmental and metabolic crystalline particle sizes and shapes e.g. CaP (0.2–1 µm size; rhomboid and prism shape), silica (1–1.5 µM size; sphere shape), TiO_2_ (80 nm size; sphere shape), cholesterol (0.2–1.5 µm size; rhomboid shape), CaOx (1–2 µm size; rhomboid and prism shape), and MSU (1–2 µm size; needle-like shape) (Fig. 1). All crystalline particles induced LDH release in the supernatant in dose dependent manner (Supplementary Figure 1). Further, when exposing these crystalline particles to human kidney (HK)-2 cells and analyzing cell death using acridine orange - propidium iodide (PI) staining, we observed that irrespective of their sizes, and shapes all crystals or crystalline particles induced cell death in HK-2 cells (Fig. 1 and Supplementary Figure 2A).

### Crystalline particles of different sizes and shapes predominately induce primary cell necrosis

**Figure 2:**
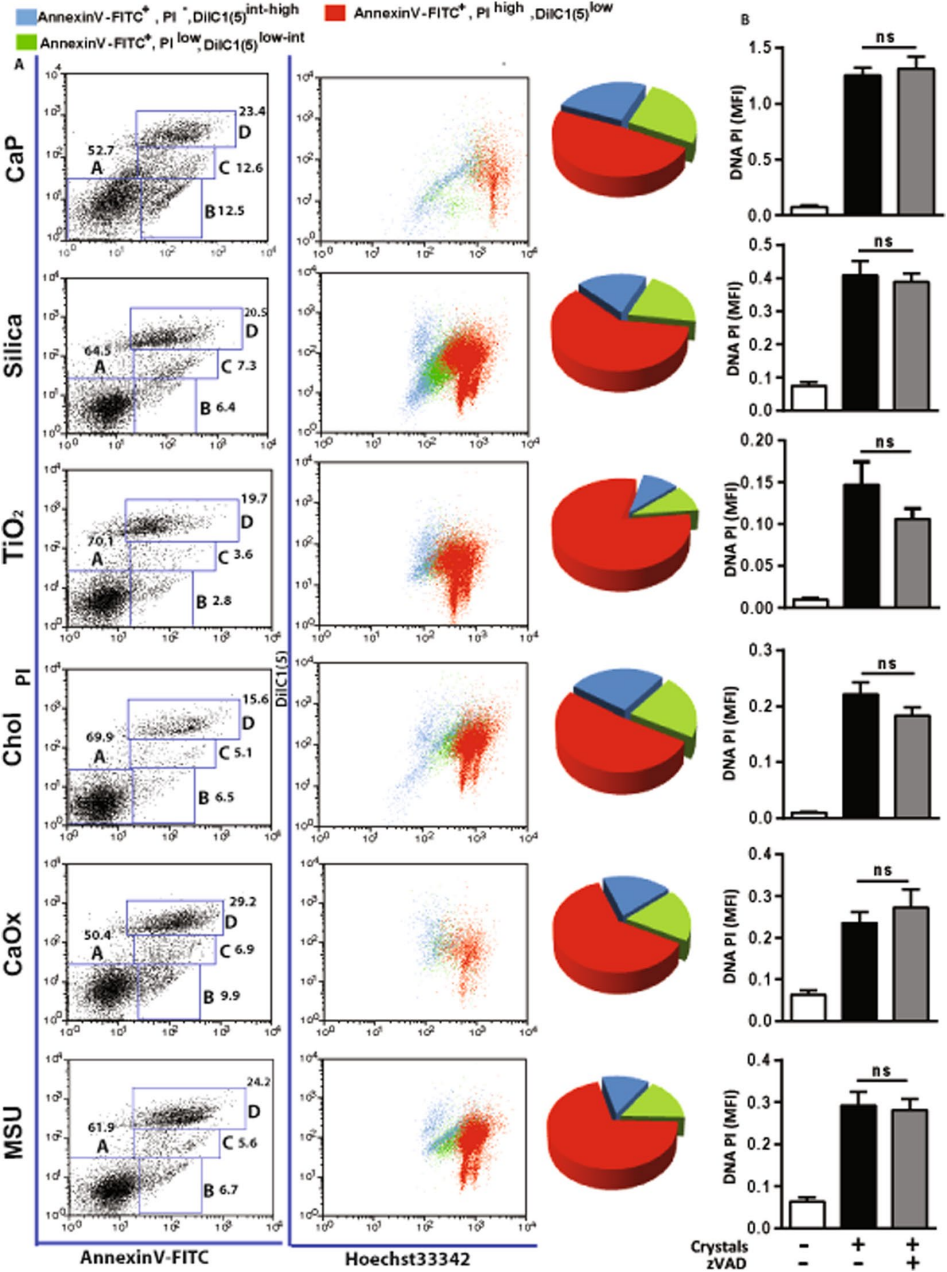
Crystals or crystalline particles induce primary necrosis in HK-2 cells. (**A**) HK-2 cells were exposed to CaP (1 mg/ml), silica (1 mg/ml), TiO2 (0.5 mg/ml), cholesterol (3 mg/ml), CaOx (1 mg/ml), and MSU (0.5 mg/ml) for 24 hrs, and different modes of cell death were analyzed by multicolor flow cytometry as described in material and methods. Note that crystals or crystalline particles mainly induce primary necrosis in HK-2 cells. Primary necrotis: AnnexinV-FITC^+^, PI^high^, DilC1(5)^low^, Secondary necrosis: AnnexinV-FITC^+^, PI^low^, DilC1(5)^low-int^, and Apoptosis: AnnexinV-FITC^+^, PI^−^, DilC1(5)^int-high^. (**B**) HK-2 cells were pretreated with a pan-caspase inhibitor zVAD-FMK (10 µM) for 30 min before crystals exposure. Cell death was analyzed after 24 hrs by quantifying DNA-PI mean fluorescence intensity (MFI). Data are expressed as mean ± SEM from three independent experiments.

To unravel the mechanisms of crystalline particle-induced cell death we performed flow cytometry and determined the type of cell death according to the positivity of Hoechst 33342, annexin V-FITC, 1,1′-dioctadecyl-3,3,3′,3′-tetramethyl-indocarbocyanine perchlorate (DiLC1) or PI. We found that environmental and metabolic crystalline particles of different sizes and shapes predominately induce primary necrosis (AnnexinV-FITC^+^, PI^high^, DilC1(5)^low^) in HK-2 cells (Fig. 2A). Secondary necrotic cells were identified as AnnexinV-FITC^+^, PI^low^, DilC1(5)^low-int^ and apoptotic cells as AnnexinV-FITC^+^, PI^−^, DilC1(5)^int-high^ (Fig. 2A). Furthermore, pre-treatment of HK-2 cells with a pan-caspase inhibitor zVAD-FMK did not reduce the DNA-PI mean florescence intensity after exposure to crystalline particles (Fig. 2B and Supplementary Figure 2B). This suggests that caspases-mediated necrosis mechanisms are not predominant forms of cytotoxicity of crystalline particles. Together, we conclude that environmental and metabolic crystalline particles predominately induce primary cellular necrosis independent of caspases.

### Three inhibitors of necroptosis consistently abrogate cytotoxicity of crystalline particles

**Figure 3:**
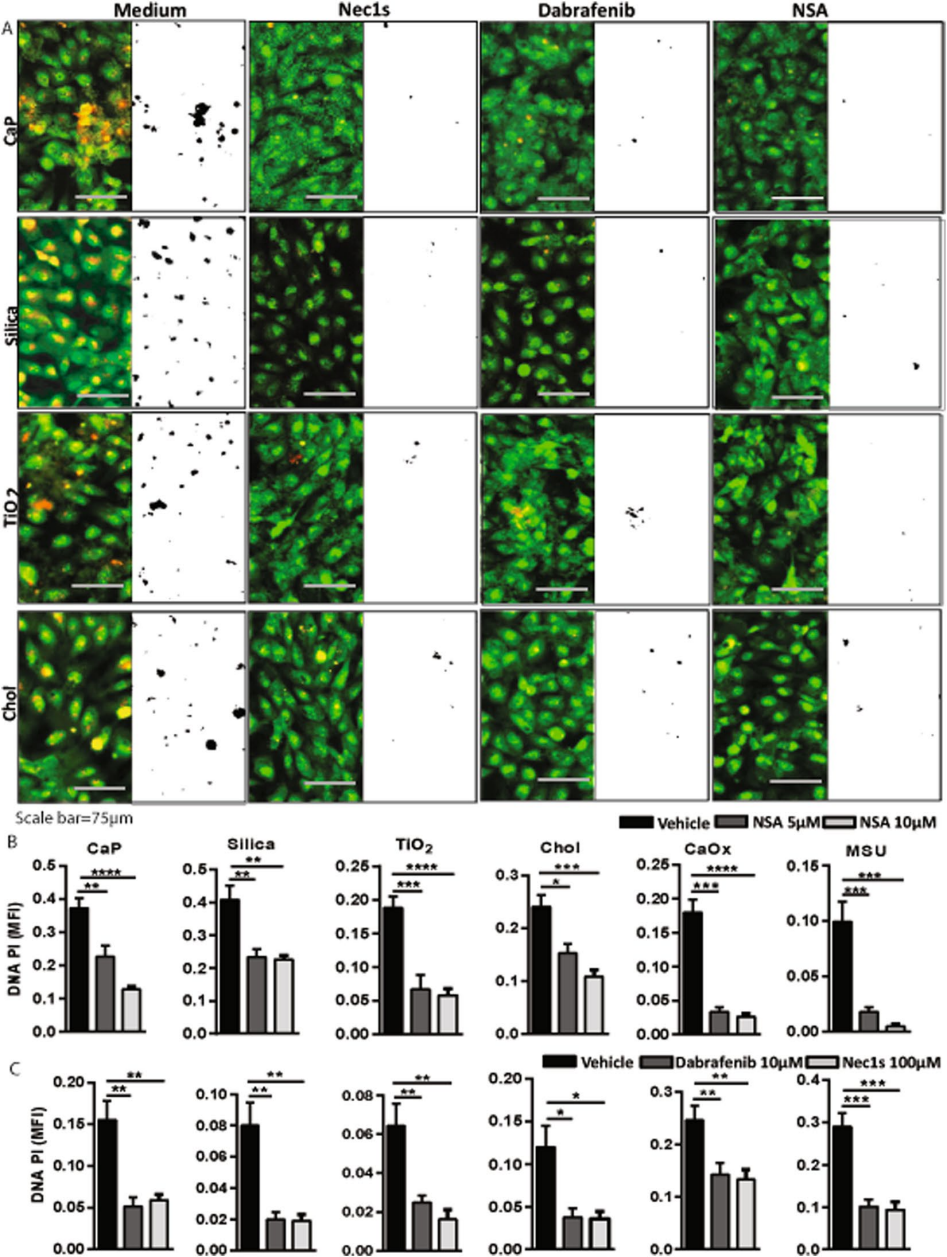
Nec1s, dabrafenib, and NSA pretreatment partially inhibit crystal cytotoxicity in HK-2 cells. HK-2 cells were pretreated with Nec1s (100 μM), dabrafenib (10 μM) or NSA (5 μM and 10 μM) 30 min before exposing to CaP (1 mg/ml), silica (1 mg/ml), TiO_2_ (0.5 mg/ml), cholesterol (3 mg/ml) for 24 hrs. (**A**) Cell death was visualized by PI stain (red color) and Acridine orange (green color). PI images were converted into black and white image for better visualization using ImageJ software. (**B**,**C**) Quantification of DNA-PI mean fluorescence intensity (MFI). Data are expressed as mean ± SEM from three independent experiments.

We have reported that crystals of CaOx and MSU induce tubular cell necroptosis during acute oxalate nephropathy^8^. Therefore, to test whether CaP-, Silica-, TiO_2-_, and cholesterol-induced cell necrosis also involves the necroptosis key proteins RIPK3, and MLKL in HK-2 cells, we first checked the expression of these proteins after exposure to crystals. We observed that exposure of crystals induced the expression of RIPK3, however, did not affect the expression of MLKL in HK-2 cells (Supplementary Figure 3). To further validate the whether CaP-, Silica-, TiO_2-_, and cholesterol-induced cell necrosis also involves the necroptosis we pre-treated HK-2 cells either with the RIPK1 inhibitor necrostatin (Nec)-1s, the RIPK3 inhibitor dabrafenib other MLKL inhibitor necrosulphonamide (NSA) and determined cell death via PI staining and LDH release. Pre-treatment with all aforementioned necroptosis signaling inhibitors partially protected HK-2 cells from CaP-, silica-, TiO_2_-, cholesterol-, CaOx-, and MSU-induced cell necrosis although different assays revealed different degrees of protection (Fig. 3A,B and Supplementary Figure 4). Nevertheless, we conclude that the cytotoxicity induced by environmental and metabolic crystals partially involves RIPK1-RIPK3-MLKL-mediated necroptosis as a regulated form of cell death.

### RIPK3 knockdown and MLKL deficiency inhibits cytotoxicity of crystalline particles

**Figure 4:**
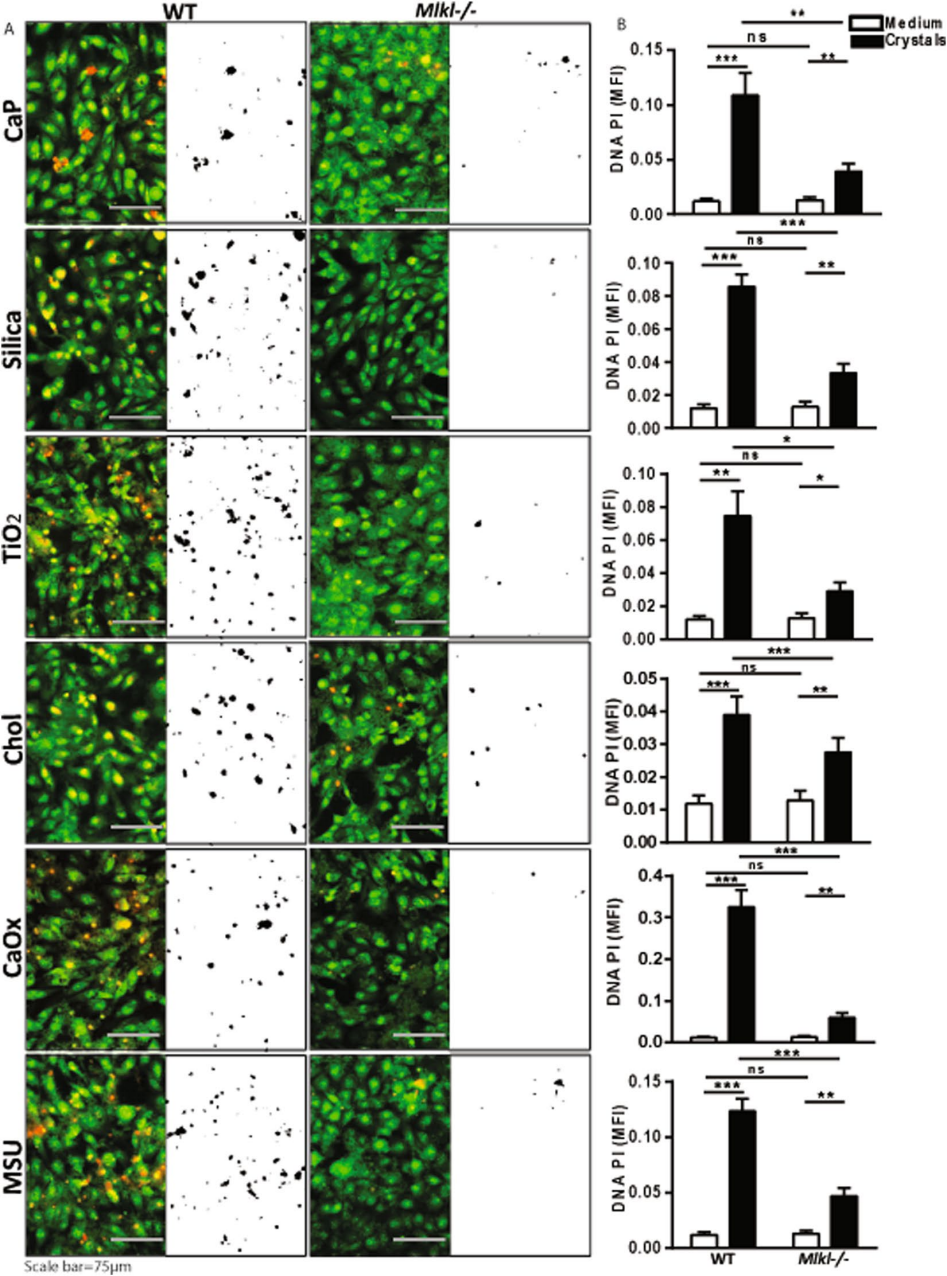
MLKL deficiency inhibits crystal cytotoxicity in murine primary tubular epithelial cells. Murine primary tubular epithelial cells (pTECs) were isolated from wild-type and *Mlkl*-deficient mice and exposed to CaP (1 mg/ml), silica (1 mg/ml), TiO_2_ (0.5 mg/ml), cholesterol (3 mg/ml), CaOx (1 mg/ml), and MSU (0.5 mg/ml) for 24 hrs. (**A**) Cell death was visualized by PI stain (red color) and Acridine orange (green color). PI images were converted into black and white image for better visualization using ImageJ software. (**B**) Quantification of DNA-PI mean fluorescence intensity (MFI). Data are expressed as mean ± SEM from three independent experiments.

Although inhibitors of necroptosis signaling e.g. Nec1s, dabrafenib, and NSA ameliorated cytotoxicity of environmental and metabolic crystalline particles in human cells, potential off-target effects of these compounds require concept validation using a second experimental approach. We knocked down the expression of RIPK3 in HK-2 cells and used primary tubular epithelial cells from *Mlkl-* deficient mice to block necroptosis. We exposed the HK-2 cells transfected with siRNA for RIPK3 and *Mlkl-* deficient primary tubular epithelial cells to CaP, silica, TiO_2_, cholesterol, CaOx, MSU and measured cell death again using PI staining and LDH assay. We found that RIPK3 knockdown and *Mlkl-*deficiency partially protected murine primary tubular epithelial cells from CaP-, silica-, CaP-, cholesterol, CaOx, and MSU-induced cell necrosis confirming the necroptosis signaling pathway to be involved in environmental and metabolic crystalline particle-induced cell death (Fig. 4 and Supplementary Figures 5–6). Interestingly, we observed different degrees of protection between various crystalline particles (Fig. 4 and Supplementary Figures 5–6). This suggests a possible impact of particle type-, size-, and shape-specific on the preferred route of regulated cell death, beyond necroptosis.

### Crystal phagocytosis is an upstream event in crystalline particle-induced cytotoxicity

**Figure 5:**
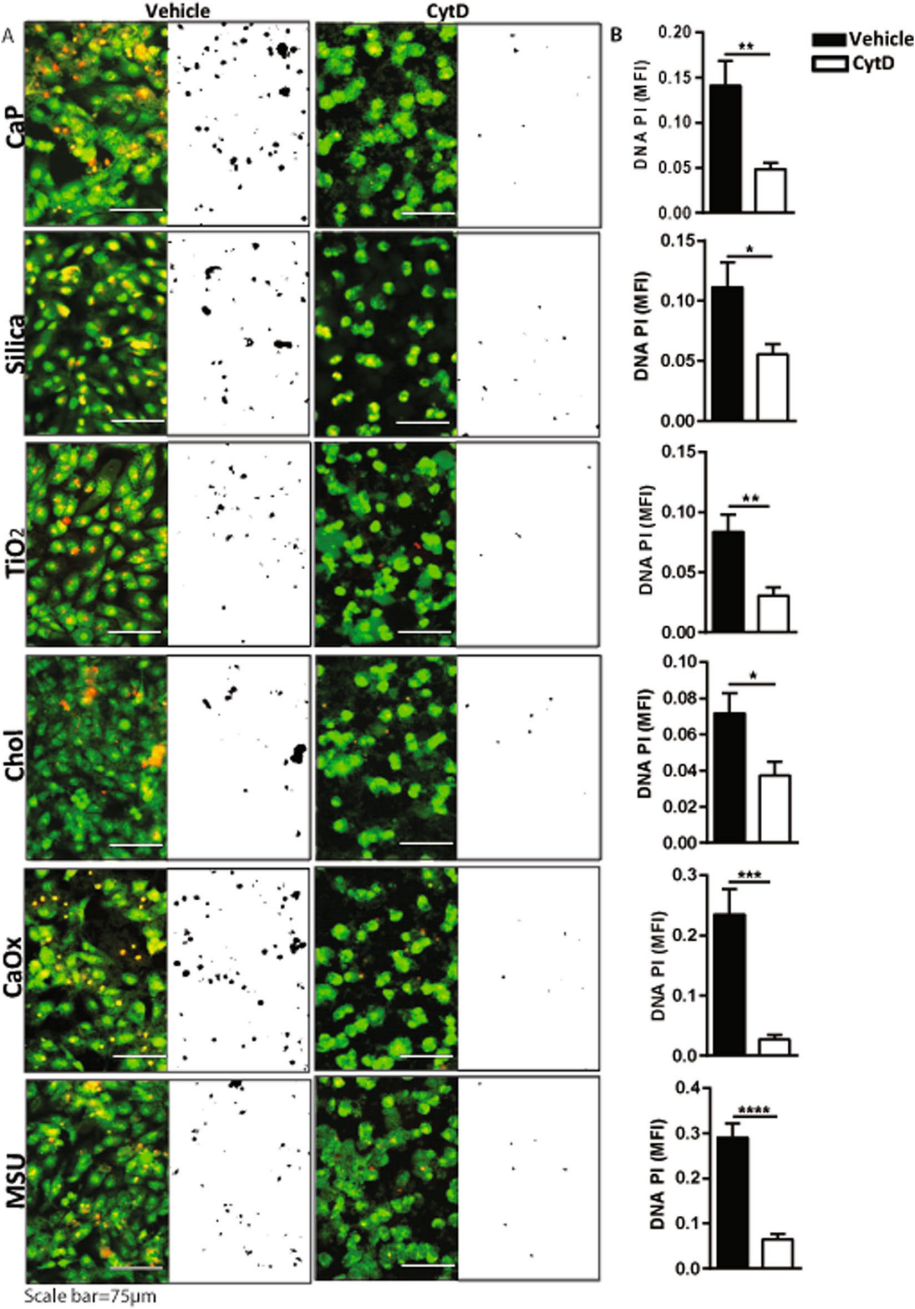
Phagocytosis of crystals or crystalline particles triggers their cytotoxicity. HK-2 cells were pretreated with the phagocytosis inhibitor cytochalasin D (Cyt D) (10 µM) 30 min before exposing to CaP (1 mg/ml), silica (1 mg/ml), TiO_2_ (0.5 mg/ml), cholesterol (3 mg/ml), CaOx (1 mg/ml), and MSU (0.5 mg/ml) for 24 hrs. (**A**) Cell death was visualized by PI stain (red color) and Acridine orange (green color). PI images were converted into black and white image for better visualization using ImageJ software. (**B**) Quantification of DNA-PI mean florescence intensity (MFI). Data are expressed as mean ± SEM from three independent experiments.

How do crystalline particles trigger RIPK1-RIPK3-MLKL signaling? As phagocytosis of CaOx crystals is an upstream event of CaOx-induced activation of the NLRP3 inflammasome^13^ we speculated on the same for crystal-induced cytotoxicity. Human kidney cells are known for their ability to phagocytose crystals^20^. To test this concept we pre-treated HK-2 cells with the cytochalasin D, an inhibitor of actin polymerization and hence phagocytosis, before exposing them to CaP, silica, TiO_2_, cholesterol, CaOx and MSU. We observed that cytochalasin D treatment prevented cytotoxicity in HK-2 cells, however, to different extents, after exposure to crystalline particles. These data suggest phagocytosis of both environmental and metabolic crystalline particles to be an essential step in crystalline particle-induced cell death (Fig. 5 and Supplementary Figure 7).

## Discussion

Crystals or crystalline particles activate the auto-amplification loop between cell death and inflammation^21^. Recent discoveries established the activation of the NLRP3 inflammasome as a central mechanism in crystal-induced inflammation^13–19^. We have reported that some crystals (e.g. CaOx) may induce NLRP3 inflammasome-independent cell death, mainly necroptosis, during acute kidney injury^8^. Here, we expand this finding by reporting a broad range of sizes and shapes of environmental and metabolic crystals or crystalline particles to induce necroptosis in human cells.

Diverse forms of crystallopathies e.g. lungs (Silica, TiO_2_), kidneys (CaP, CaOx, MSU), joints (MSU), and arteries (cholesterol) involve crystal-induced NLRP3 inflammasome-mediated inflammation leading to tissue injury^1^. The mechanisms that activate the NLRP3 inflammasome include phagocytosis of crystals and subsequent lysosomal rupture^13–19^. Although NLRP3 agonistic activities of crystals suggest that they may induce NLRP3/caspase-1 mediated pyroptosis, a form of regulated necrosis^22,23^, we observed that caspase inhibition did not prevent cytotoxicity of crystalline particles. These data suggests that crystalline-particles induced cell death involves caspase-independent mechanisms. Furthermore, flow cytometry analysis of cytotoxicity of crystalline particles revealed the involvement of primary cellular necrosis as a predominant form of crystalline-particles induced cell death. Our recent report suggests an involvement of necroptosis in oxalate crystal-induced acute kidney injury^8^. In a similar manner, depletion or deficiency of the core proteins of necroptosis signaling, RIPK3 and MLKL partially prevented crystalline particle-induced cell necrosis. Such partial protection is suggestive of the involvement of other forms of regulated necrosis pathways in crystalline particle cytotoxicity. For example, oxalate crystal-induced toxicity on tubular cells can be blocked with ferrostatin-1, a compound specifically inhibiting an iron-dependent form of cell death named ferroptosis^24^. Moreover, since the degree of protection differed between crystalline particles, it seems that the contribution of necroptosis to cytotoxicity is particle-specific. Nevertheless, our data implies both environmental, as well as metabolic, crystalline particle-induced cell death partially involves necroptosis.

RIPK1 acts as an intrinsic suppressor of necroptosis signaling^25^. Certain post-translational events, such as ubiquitination upon TNFR1-ligation, lead to abrogation in its suppressor function and formation of the necrosome consisting of RIPK1, RIPK3, and MLKL, which execute necroptosis^26^. Small molecule chemical inhibitors e.g. Nec1s keep RIPK1 in its anti-necroptotic confirmation and prevent necroptosis^25^. We observed that pretreatment of cells with Nec1s, as well as an RIPK3 inhibitor dabrafenib, prevented crystalline particle-induced cell death. Furthermore, an MLKL inhibitor NSA also prevented crystalline particle-induced cell death in a dose-dependent manner. These data further confirm the partial involvement of necroptosis in both environmental and crystalline particle-induced cell death.

Moreover, previous studies report that inflammatory responses of cells highly depend on the sizes and shapes of particles^3,27–31^. The crystals or crystalline particles we used in the present study were significantly different in their sizes, as well as shapes. Interestingly, we observed that irrespective of the sizes and shapes, all crystalline particles induced necroptosis in human cells, however, to different degrees. This suggests size- and shape-specific involvement of other modalities of cell death e.g., possibly apoptosis, ferroptosis or mitochondria permeability transition-related necrosis^32^. Furthermore, we report that inhibition of actin polymerization, a pre-requisite for phagocytosis, prevented cytotoxicity of crystalline particles implying the involvement of phagocytosis in crystalline particle-induced cell death. However, whether the actin polymerization or phagocytosis induce cellular necrosis after exposure to crystalline particles remains unclear since larger particles sizes and shapes are difficult to internalize via phagocytosis. Together, our data demonstrate that not only the kind (environmental or metabolic), but also a wide range of sizes and shapes of crystals or crystalline particles partially induce necroptosis as a regulated form of cell death.

**Figure 6:**
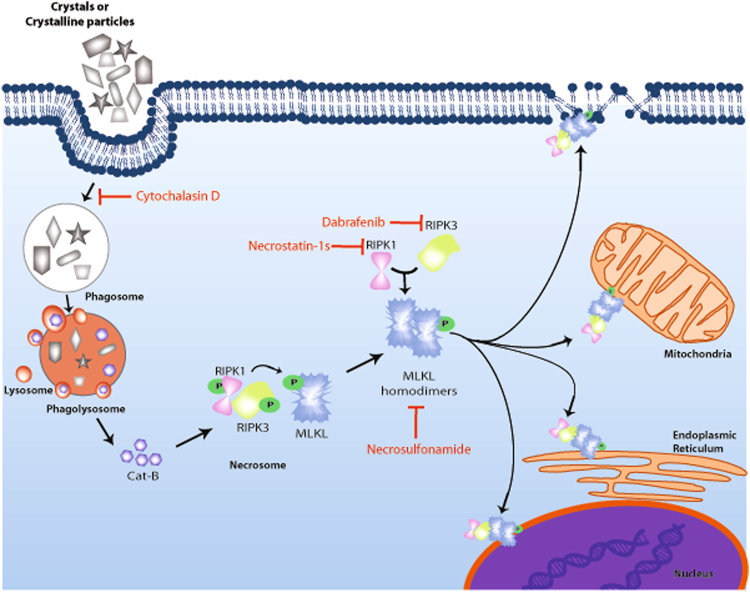
Schematic representation of molecular mechanisms of crystalline particle-induced necroptosis. After exposure, crystals or crystalline particles are phagocytized by human cells. The frustrated crystal phagocytosis leads to lysosomal destabilization and subsequent leakage of cathepsin B (or other lytic proteases) into the cytosol. This cytosolic cathepsin B binds directly to RIPK1, an indigenous inhibitor of necroptosis, and induces its degradation. The degradation of RIPK1 leads to the formation of the necrosome that consists of RIPK3 and MLKL. A series of auto/trans phosphorylation activate RIPK3 and MLKL, leading to formation of oligomers of MLKL, which then are translocated to different membranes, e.g. nucleus, endoplasmic reticulum, mitochondria and plasma membranes etc. After binding MLKL, oligomers induce pore formation in these membranes leading to necroptosis. Crystals or crystalline particle-induced necroptosis can be inhibited by blocking phagocytosis using cytochalasin D or necroptosis using RIPK1 inhibitors necrostatin-1s, RIPK3 inhibitor dabrafenib, and MLKL inhibitor necrosulfonamide. Cat-B: Cathepsin B, RIPK: Receptor interacting protein kinase, MLKL: Mixed lineage kinase domain-like.

In conclusion, a broad range of environmental and metabolic crystal sizes and shapes induce RIPK1-RIPK3-MLKL-mediated necroptosis (Fig. 6). Therefore, therapeutic blockade of this pathway using the RIPK1 stabilizer necrostatin-1s, the RIPK3 inhibitor dabrafenib, and the MLKL inhibitor necrosulphonamide may prevent crystal-induced necroinflammation, tissue damage, and subsequent organ dysfunction. These findings identify RIPK1, RIPK3, and MLKL as potential molecular targets to potentially limit tissue injuries in silicosis, TiO_2_-induced lung injury, cholesterol embolism, oxalate-/phosphate-/urate-nephropathy, gout, as well as other crystallopathies.

## Material and Methods

### Cell culture studies

Human kidney cells (HK2) were maintained in DMEM medium (GIBCO, Invitrogen, CA, USA) with 10% fetal calf serum (FCS) and 1% penicillin-streptomycin (PS). HK-2 cell line was originally purchased from ATCC. Primary tubular epithelial cells (pTECs), isolated from the kidney of wild-type or *Mlkl-*deficient mice kindly provided by James Murphy, Parkville, Australia^33^, were maintained in DMEM/F12 medium with 10% FCS, 1% PS, 125 ng/ml prostaglandin E1 (Calbiochem, Germany), 25 ng/ml EGF, 1.8 mg/ml l-thyroxine, 3.38 ng/ml hydrocortisone and 2.5 mg/ml of insulin–transferrin—sodium selenite supplement (all from Sigma-Aldrich, Germany)^8,34^. All cells were stimulated with crystals of CaP 1 mg/ml (0.2–1 µm size; rhomboid and prism shape; Santa Cruz Biotechnology, Germany), silica 1 mg/ml (1–1.5 µM size; sphere shape; Alfa aesar, Germany), TiO_2_ 0.5 mg/ml (80 nm size; sphere shape; Io-li-Tec, Germany), cholesterol 3 mg/ml (0.2–1 µm size; rhomboid shape; Sigma, Germany), CaOx 1 mg/ml (1–2 µm size; rhomboid and prism shape; Alfa aesar, Germany) and MSU 0.5 mg/ml (needle like shape, 1–2 µm size; Invivogen, Germany). Cells were pretreated with cytochalasin D (10 µM, Sigma, Germany), NSA (5 or 10 µM, Millipore, Germany), dabrafenib (10 µM, Selleckchem, Germany) or Nec-1s (100 µM, Bio-vision, Milpitas, CA, USA) 30 min before exposing to crystals.

### Cell death assays

Dead cells were identified using acridine orange and PI stain (Thermo fisher scientific, Germany). Acridine orange stained cells in green whereas PI stained dead cells in red. Fluorescence signals were detected using Leica fluorescence microscope (Leica, Germany), and quantified using ImageJ software (USA). In addition, cell death was also evaluated using Lactate dehydrogenase (LDH) cell cytotoxicity assay kit (Roch, Germany) according to manufacturer’s protocol.

### Transmission Electron Microscopy (TEM)

One drop of suspension of crystals in PBS transferred onto formvar coated copper grids (Plano, Wetzlar, Germany). Excessive liquid was removed after 30 second and grids allowed to air-dry. Crystals were viewed using a JEOL EXII 1200 transmission electron microscope (Jeol, Tokyo, Japan) at 80 kv. KeenViewII (Olympus, Germany) digital camera used to take pictures and processed by the iTEM software package (analySISFive, Olympus, Germany).

### Multi-parameter classification of cell death by flow cytometry

Cell death was induced in HK-2 cells (300000 cells per well) by exposing them to different crystals and was characterized by analyzing cytofluorometric parameters (size, granularity, PS exposure, plasma membrane integrity, mitochondrial membrane potential and DNA content)^35^. Briefly, collected cells were incubated for 30 min at room temperature with 400 μl of freshly prepared four-colour staining solution (1.8 μg/ml AxA5-FITC, 100 ng/ml PI and 10 nM DiIC1(5), 1ng/ml Hoechst 33342) in Ringer’s solution. Flow cytometry was performed with a FACSCantoII flow cytometer (BD, Germany). Data analysis was performed with FlowJo software (USA). Cells were classified according to their location in the forward scatter (FSc; size) versus side scatter (SSc; granularity) dot plot and their staining patterns analyzed as described^8^. In brief, cells were considered primary necrotic if they express AnnexinV-FITC^+^, PI^high^, DilC1(5)^low^, secondary necrotic were AnnexinV-FITC^+^, PI^low^, DilC1(5)^low-int^ and apoptotic cells were identified as AnnexinV-FITC^+^, PI^−^, DilC1(5)^int-high^. To assure equal numbers of cells, we recorded 100000 events per sample. The basic of the gating strategy was sideward scatter vs. forward scatter, and then AnnexinV vs. PI staining. The AnnexinV/PI staining allowed us to differentiate between the cell populations that were then further characterized using the DilC1(5) and Hoechst33342 dyes.

### siRNA experiments

HK-2 cells were transfected with 30 nM of three unique 27mer siRNA duplexes targeting RIPK3 (SR307548), and scrambled siRNA (SR30004) (ORiGene, USA) using Neon transfection system (ThermoFisher, Germany). After 48 hrs, transfected cells were exposed to CaP 1 mg/ml, silica 1 mg/ml, TiO_2_ 0.5 mg/ml, cholesterol 3 mg/ml, CaOx 1 mg/ml and MSU 0.5 mg/ml for 24 h. Cell death was analysed by prodidium iodide positivity and LDH assays. Cell viability was analysed using acridine orange assay.

### RNA preparation and real-time quantitative PCR

Total RNA were extracted from transfected cells using a Qiagen RNA extraction kit (Qiagen, Germany) following the manufacturer’s instructions. From isolated RNA, cDNA was prepared using reverse transcriptase (Superscript II; Invitrogen, USA). Real-time reverse transcription PCR was performed using SYBRGreen PCR master mix and was analysed with a Light Cycler 480 (Roche, Germany). All gene expression values were normalized using 18s rRNA as a house keeping gene. All primers used for amplification were from Metabion (Martinsried, Germany). The following primer has been used to check RIPK3 expression after transfection: RIPK3: 5′-CATGGAGAACGGCTCCTTGT-3′ (for), 5′-GGTTCTGGTCGTGCAGGTAA-3′ (rev).

### Protein isolation and immunoblotting

Proteins from cells were isolated using RIPA lysis buffer (Tris-HCl 50 mM, NaCl 150 mM, sodium orthovanadate 100 mM, sodium deoxycholate 0.5%, NP40 4%, Triton X-100 2%, EDTA 5 mM, sucrose 300 mM, and Roche protease inhibitors). After measuring protein concentrations, 10 mg of protein was mixed with 5 sodium dodecyl sulfate loading buffer (100 mmol/l Tris-HCl, 4% sodium dodecyl sulfate, 20% glycerol and 0.2% bromophenol blue) for western blot analysis. Samples were heated at 95° C for 5 min. Proteins were separated by sodium dodecyl sulfate-polyacrylamide gel electrophoresis and then transferred to a polyvinylidene difluoride membrane. Nonspecific binding to the membrane was blocked for 1hr at room temperature with 5% bovine serum albumin in tris-buffered saline buffer (20 mmol/l Tris-HCl, 150 mmol/l NaCl and 0.1% Tween 20). The membranes were then incubated overnight at 4°C with the following primary antibodies: RIPK3 (1:250, Abcam, UK), anti-human total MLKL (1:250, R&D systems, Germany) and β-actin (1:1000, Cell Signalling, USA), followed by incubation with secondary antibody anti-biotin or anti-rabbit IgG labelled with HRP. Immunostained bands were detected using a chemiluminescence kit (ECL kit, GE Healthcare, UK).

### Statistical analysis

Data are presented as mean ± SEM. A comparison of groups was performed using paired students t-test or one-way ANOVA with posthoc Bonferroni’s correction was used for multiple comparisons. A value of p < 0.05 was considered to indicate statistical significance. Graphpad Prism 5 was used for statistical data analysis.

## Electronic supplementary material

Supplementary information
