# Supplementary material for: Phagocytosis of environmental or metabolic crystalline particles induces cytotoxicity by triggering necroptosis across a broad range of particle size and shape
Source: Sci Rep. 2017 Nov 14;7:15523. doi: 10.1038/s41598-017-15804-9 (PMC5686194; doi:10.1038/s41598-017-15804-9)
Supplement: Supplementary file 1 — Supplementary information [file 41598_2017_15804_MOESM1_ESM.pdf]

Phagocytosis of environmental or metabolic crystalline particles induces cytotoxicity by triggering necroptosis across broad range of particle size and shape

Mohsen Honarpisheh[1], Orestes Foresto-Neto[1], Jyaysi Desai[1], Stefanie Steiger[1], Lidia Anguiano Gómez[1], Bastian Popper[2], Peter Boor[3], Hans-Joachim Anders[1*], Shrikant R. Mulay[1*]

1 Medizinische Klinik und Poliklinik IV, Klinikum der Universität, München, Munich, 80336, Germany
2 Biomedical Center (BMC), Department for Cell Biology, Ludwig-Maximilians University, Munich, 82152, Germany
3 Institute of Pathology & Dept. of Nephrology, University Clinic of RWTH Aachen, Aachen 52074, Germany

Supplementary information

Supplementary figure 1

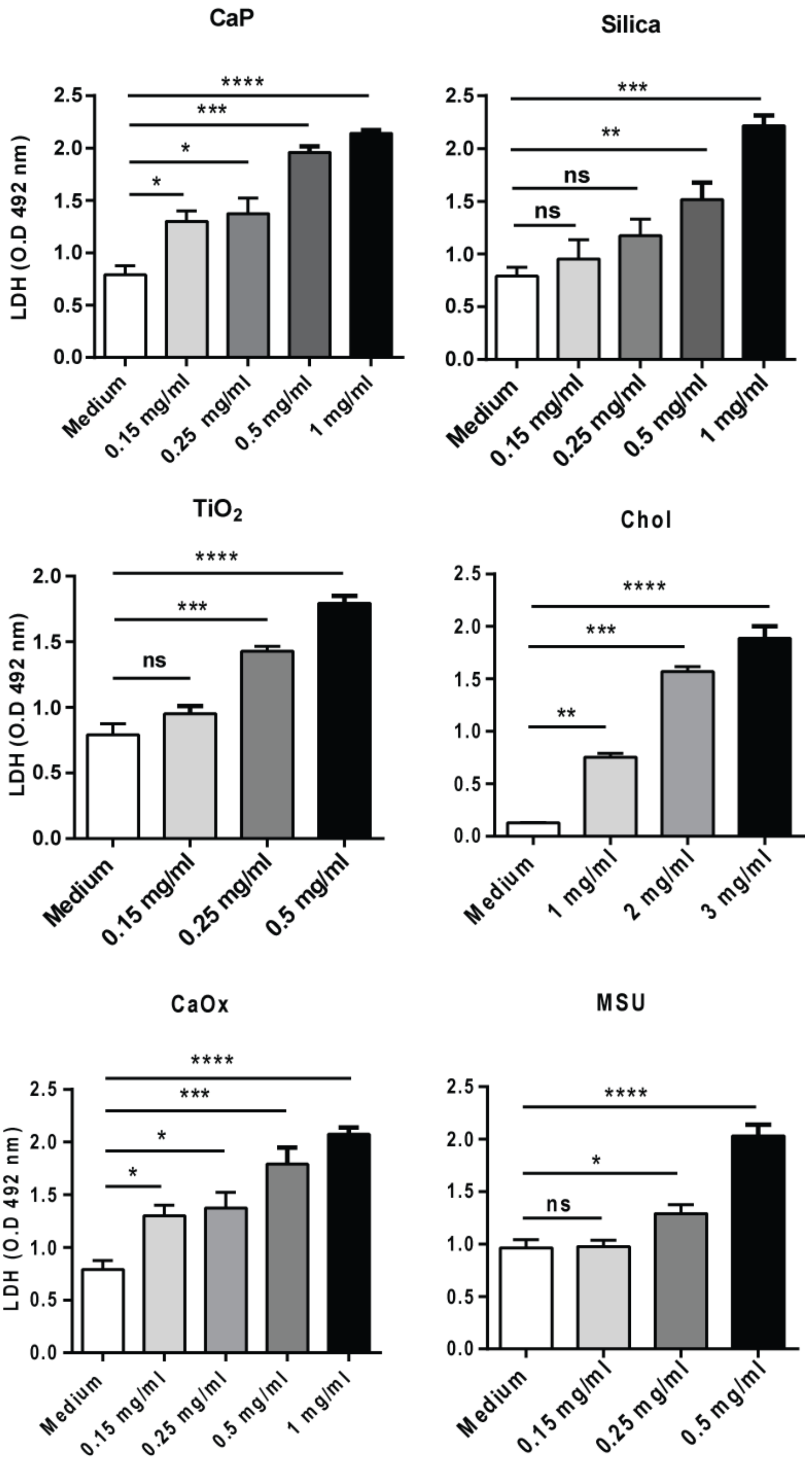

Supplementary figure 1.

HK-2 cells were exposed different doses of crystals and cell death was analyzed after 24 hrs by quantifying LDH release in the supernatant. Data are expressed as mean ± SEM from three independent experiments.

Supplementary figure 2

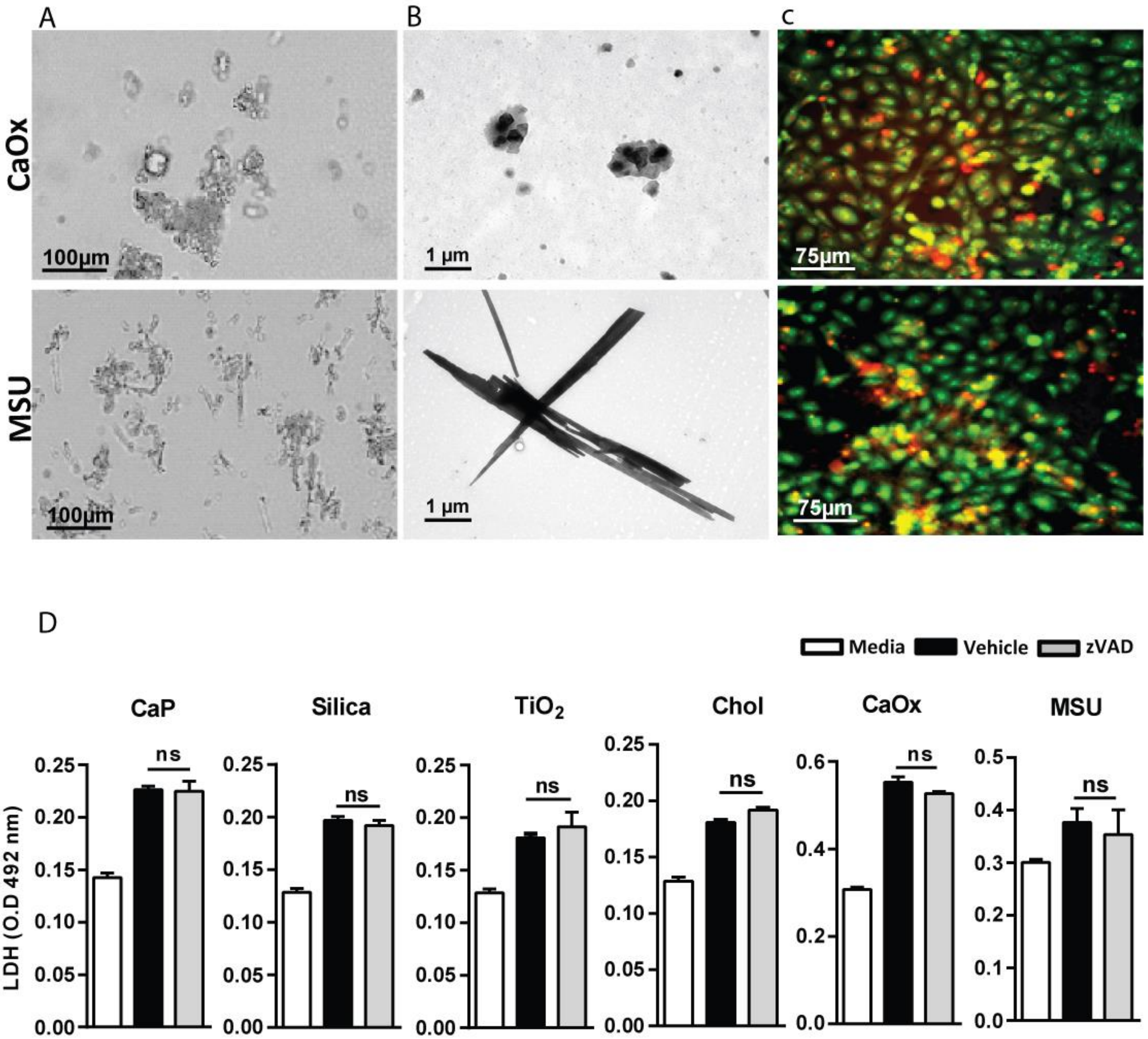

Supplementary figure 2.

A-B. Crystals of CaOx and MSU were visualized by light microscopy (A), and TEM (B). Note the different sizes and shapes of all crystals. C. HK-2 cells were exposed to CaOx (1 mg/ml) and MSU (0.5 mg/ml) for 24 hrs. Cell death was visualized by PI stain (red color). Acridine orange (green color) stained live cells. PI images were converted into black and white image for better visualization using ImageJ software. D. HK-2 cells were pretreated with the pan-caspase inhibitor zVAD-FMK (10 µM) for 30 min before exposing to CaP (1 mg/ml), silica (1 mg/ml), TiO2 (0.5 mg/ml), cholesterol (3 mg/ml), CaOx (1 mg/ml), and MSU (0.5 mg/ml). Cell death was analyzed after 24 hrs by quantifying LDH release in the supernatant. Data are expressed as mean ± SEM from three independent experiments.

Supplementary figure 3

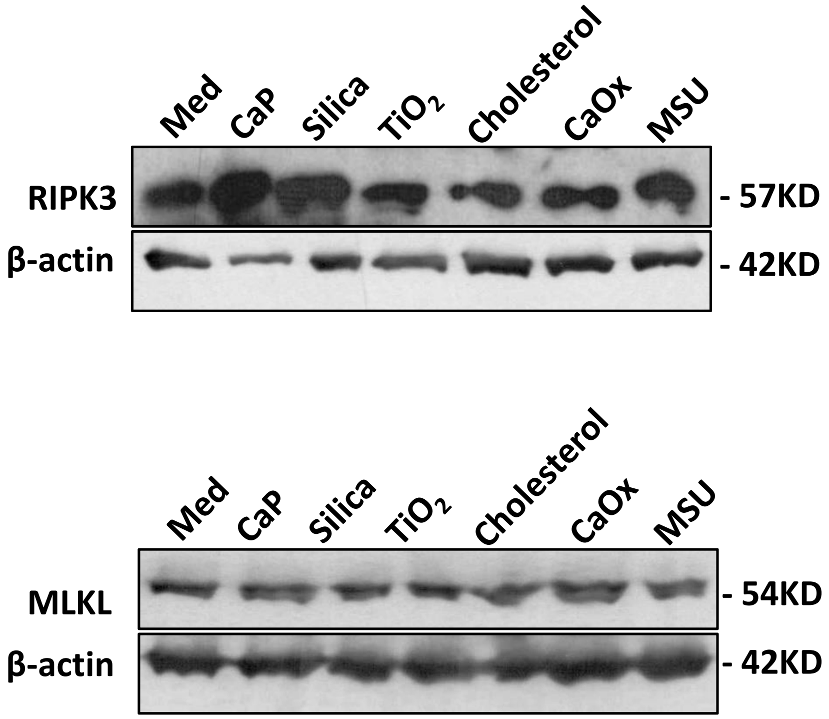

Supplementary figure 3.

Protein expression of RIPK3 and MLKL was determined by western blot from total proteins isolated 18h after stimulation of HK-2 cells with CaP (1 mg/ml), silica (1 mg/ml), TiO2 (0.5 mg/ml), cholesterol (3 mg/ml), CaOx (1 mg/ml), and MSU (0.5 mg/ml). β-actin was used as loading control.

Supplementary figure 4

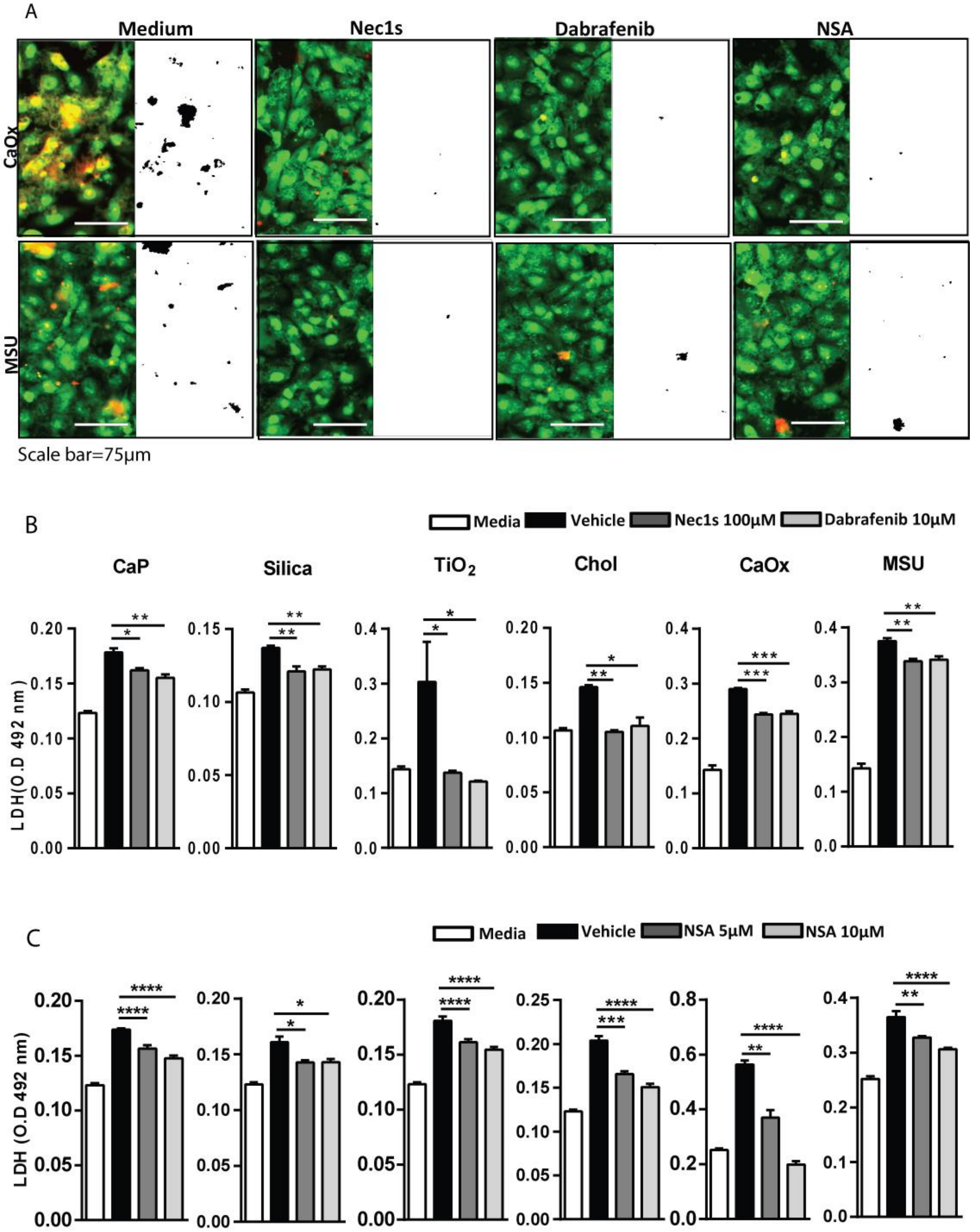

Supplementary figure 4.

A. HK-2 cells were pretreated with Nec1s (100 μM), dabrafenib (10 μM) or NSA (5 μM and 10 μM) 30 min before exposing to CaOx (1 mg/ml) and MSU (0.5 mg/ml) for 24 hrs. Cell death was visualized by PI stain (red color) and Acridine orange (green color). PI images were converted into black and white image for better visualization using ImageJ software. B-C. HK-2 cells were pretreated with Nec1s (100 μM), dabrafenib (10 μM) or NSA (5 μM and 10 μM) for 30 min before exposing to CaP (1 mg/ml), silica (1 mg/ml), TiO_2 (0.5 mg/ml), cholesterol (3 mg/ml), CaOx (1 mg/ml), and MSU (0.5 mg/ml). Cell death was analyzed after 24 hrs by quantifying LDH release in the supernatant. Data are expressed as mean ± SEM from three independent experiments.

Supplementary-Fig5

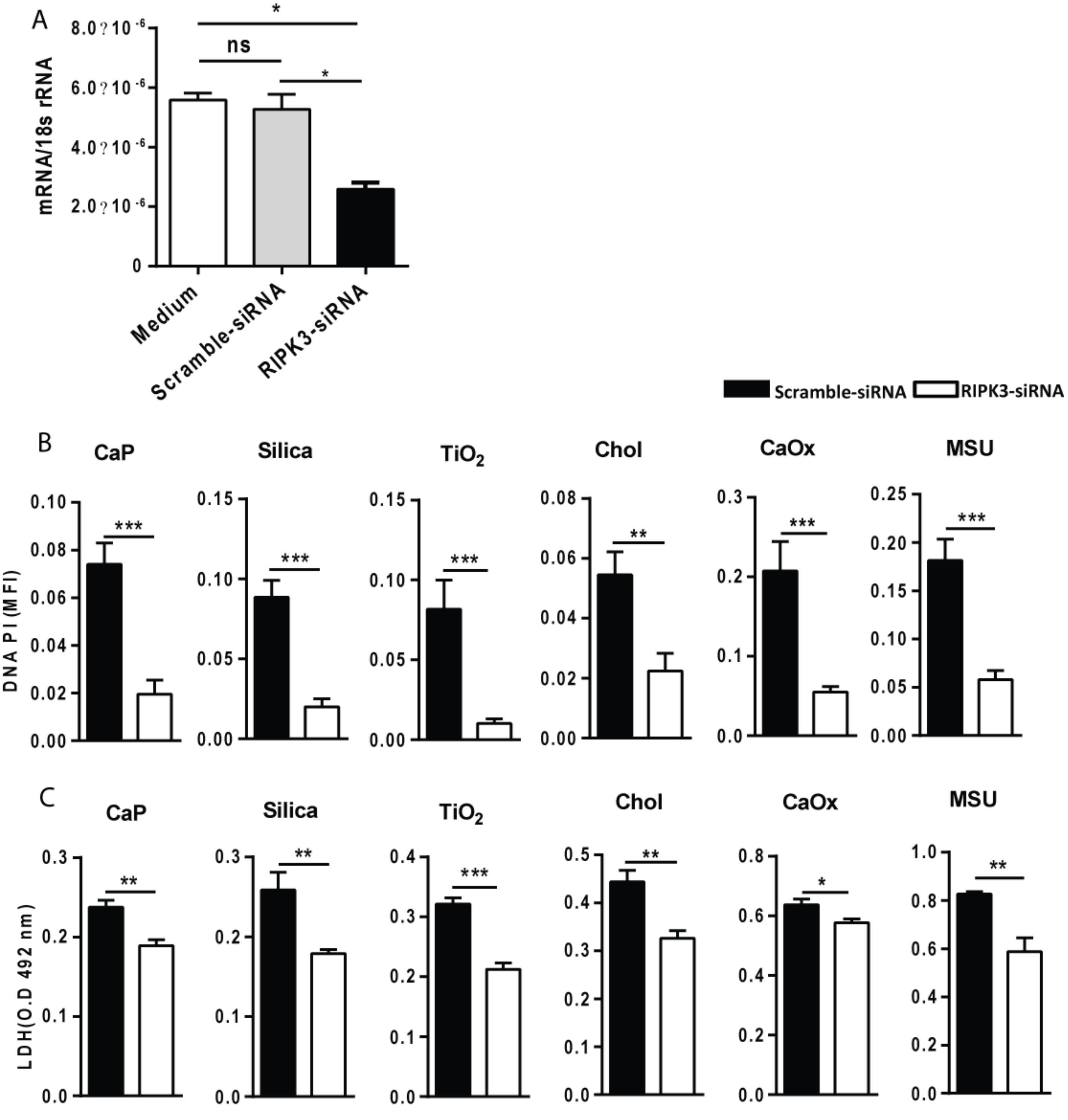

Supplementary figure 5.

A-B. HK-2 cells were transfected with specific small inhibitor (si) RNA for RIPK3 or a control siRNA of scrambled sequence. The efficiency of knockdown was determined by RT-PCR for RIPK3 (A). Transfected cells were exposed CaP (1 mg/ml), silica (1 mg/ml), TiO2 (0.5 mg/ml), cholesterol (3 mg/ml), CaOx (1 mg/ml), and MSU (0.5 mg/ml). Cell death was assessed by quantifying PI positivity (B) and by quantifying LDH release in the supernatant (C) 24h later. Data are expressed as mean ± SEM from three independent experiments.

Supplementary figure 6

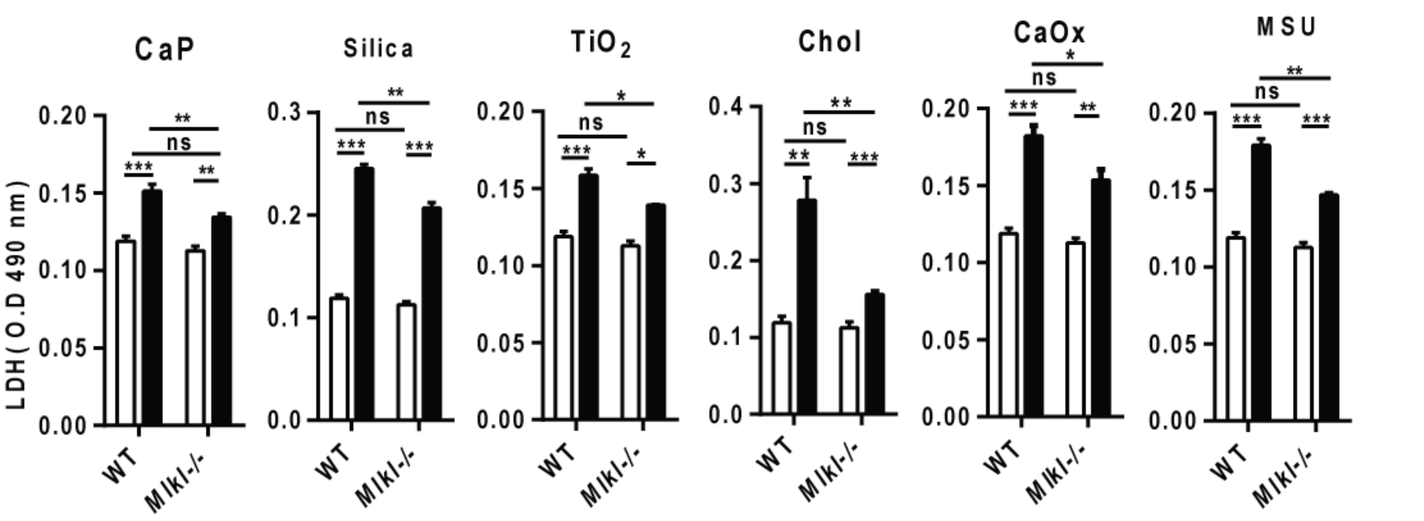

Supplementary figure 6.
Primary tubular epithelial cells (pTECs) were isolated from wild-type and *Mlkl*-deficient mice and exposed to CaP (1 mg/ml), silica (1 mg/ml), TiO2 (0.5 mg/ml), cholesterol (3 mg/ml), CaOx (1 mg/ml), and MSU (0.5 mg/ml). Cell death was analyzed after 24 hrs by quantifying LDH release in the supernatant. Data are expressed as mean ± SEM from three independent experiments.

Supplementary figure 7

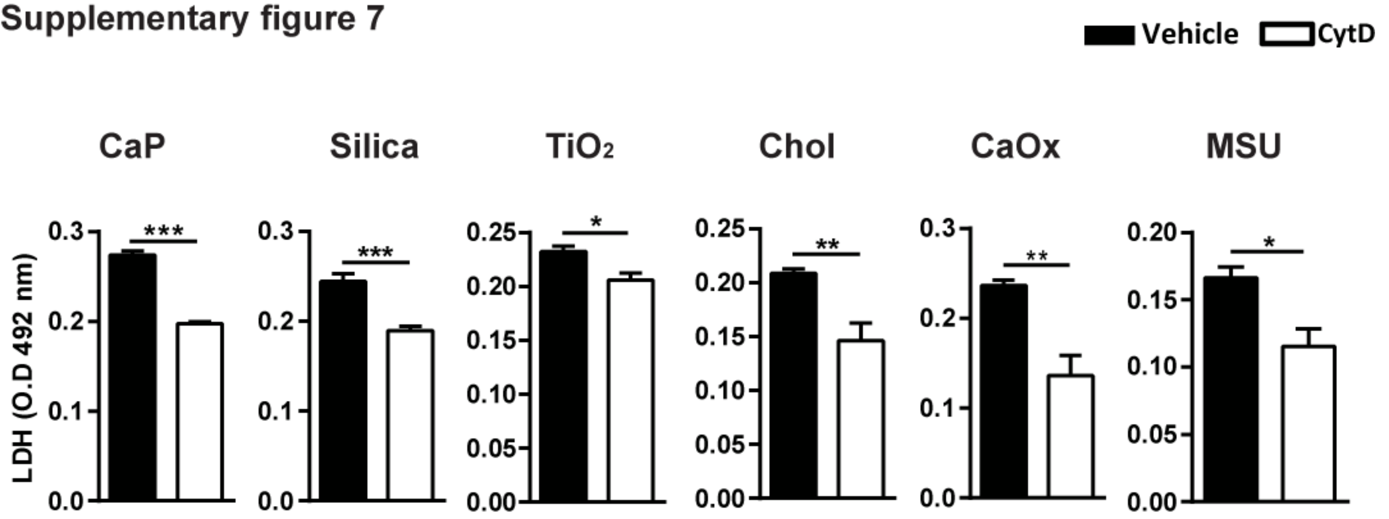

Supplementary figure 7.

HK-2 cells were pretreated with the phagocytosis inhibitor cytochalasin D (Cyt D) (10 μM) 30 min before exposing to CaP (1 mg/ml), silica (1 mg/ml), TiO_2 (0.5 mg/ml), cholesterol (3 mg/ml), CaOx (1 mg/ml), and MSU (0.5 mg/ml) for 24 hrs and LDH release in the supernatant was measured. Data are expressed as mean ± SEM from three independent experiments.
